# Comparison of clinical features in pathologically confirmed PSP and MSA patients followed at a tertiary center

**DOI:** 10.1038/npjparkd.2015.7

**Published:** 2015-05-21

**Authors:** Tao Xie, Un J Kang, Sheng-Han Kuo, Markos Poulopoulos, Paul Greene, Stanley Fahn

**Affiliations:** 1 Department of Neurology, University of Chicago Medicine, Chicago, IL, USA; 2 Neurological Institute, Columbia University Medical Center, New York, NY, USA; 3 Department of Neurology, University of Vermont and Eastern Maine Medical Center, Burlington, VT, USA; 4 Department of Neurology, Mount Sinai Hospital, New York, NY, USA

## Abstract

**Background/Objectives:**

The clinical diagnosis of progressive supranuclear palsy (PSP) and multiple system atrophy (MSA) remains challenging due to heterogeneity of the diseases.

**AIMS:**

Here we compared the clinical features of PSP and MSA to gain insight into their diagnosis and prognosis, particularly the prognostic value of down-gaze palsy latency in PSP progression.

**Methods:**

We reviewed clinical features of pathologically confirmed 10 PSP and 13 MSA patients, incidentally matched in age-at-onset, gender, and disease duration, followed at Columbia University Medical Center during 1994–2009.

**Results:**

The final antemortem diagnosis was incorrect in 30% of PSP (all lacking down-gaze palsy) and 23% of MSA patients. Falls in the first year of the disease, pyramidal involvement and freezing of gait during the course were similar between PSP and MSA. Ataxia and apraxia were in 50% of the PSP patients. Parkinsonism responsive to levodopa treatment was in 30% of the PSP (all with resting tremor) and 50% of the MSA patients. Dysautonomia in MSA could occur as early as 3 years preceding the motor symptoms, with 46% within the first year of the motor onset, but 15% did not have dysautonomia in life. The latency of down-gaze palsy and urogenital dysfunction and MMSE scores at first visit in PSP, and the latency of falls and wheelchair confinement in MSA were all associated with the disease progression.

**Conclusions:**

We confirmed most of the previously published characterizations, and identified for the first time the prognostic value of down-gaze palsy latency in PSP progression.

## Introduction

The diagnosis of progressive supranuclear palsy (PSP) and multiple system atrophy (MSA) could be very challenging in the absence of characteristic symptoms and signs or the presence of atypical ones due to the heterogeneity of the diseases. Clinically, the diagnostic hallmarks of PSP are the presence of down-gaze palsy and frequent falls in the first year of the disease onset.^1–3^ The absence of down-gaze palsy often leads to misdiagnosis of PSP in as high as 60% of the pathologically confirmed patients.^4–6^ The presence of atypical symptoms in the ‘exclusion criteria’ of PSP, such as apraxia typically seen in corticobasal degeneration, or dysautonomia and ataxia typically seen in MSA,^2^ could make the diagnosis of PSP even more difficult. The presence of dysautonomia is a prerequisite for the diagnosis of MSA, with dominant parkinsonism for MSA-P and cerebellar ataxia for MSA-C.^7^ The absence of the dysautonomia could similarly make the diagnosis of MSA very difficult.

Studies on clinical features in pathologically verified PSP cases greatly advanced our knowledge on PSP.^8–11^ PSP was classified into PSP-RS (Richardson-Steele –Olszewski syndrome, 54–63%) and PSP-P (parkinsonism, 26–32%) as the major two types, besides other types including the unclassified one with the features of both PSP-RS and PSP-P, pure akinesia with gait freezing, corticobasal syndrome, and progressive non-fluent aphasia.^8,10,11^ An ataxia type was also proposed.^12^ PSP-RS is clinically defined as the presence of down-gaze palsy, falls and cognitive dysfunction in the first 2 years of the disease,^8,10^ with more rapid progression and shorter disease duration than PSP-P, which has bradykinesia, tremor and some response to parkinsonian medication.^8,10^ The pathological basis of these different clinical types is due to the difference in brain areas and severity involved by the tauopathy, with more severe and widespread pathology in PSP-RS than PSP-P and subtle difference in the amount of insoluble tau protein between them as well.^9^ The potential prognostic role of the down-gaze palsy latency in the clinical progression of the PSP, though very important, has rarely been studied before.

Here we compare the clinical features and the evolution of the clinical hallmarks of down-gaze palsy in PSP and dysautonomia in MSA and other important symptoms, and their relationship with the disease duration in pathologically confirmed patients who happened to be matched in age-at-onset (AAO), gender, and disease duration. They had years of follow-up in a single tertiary movement disorder center with methodological and expertize uniformity. This study would help a better diagnostic and prognostic assessment of PSP and MSA, in particular for the first time, the prognostic value of down-gaze palsy latency in PSP progression.

## Materials and methods

### Pathologically confirmed cases

We reviewed all the charts in patients with PSP or MSA who had been following up during 1994–2009 in the Center for Parkinson’s Disease and Other Movement Disorders at Columbia University Medical Center with autopsy confirmed diagnosis. The neuropathological diagnostic criteria for PSP and MSA were as described before by our center.^13,14^ In brief, the neuropathological diagnosis of PSP includes (1) the presence of tau-positive tufted astrocytes in the cerebral cortex, neostriatum, and amygdaloid nucleus; (2) globose neuronal tangles in at least seven of the nine following sites: cerebral cortex, neostriatum, globus pallidus, subthalamic nucleus, red nucleus, pars compacta of the substantia nigra, pontine nuclei, inferior olivary nucleus, and dentate nucleus of the cerebellum; and (3) scattered tau-positive glial cytoplasmic inclusions. Glial cytoplasmic inclusions, which involve oligodendrocytes, are the molecular hallmark of MSA. They are labeled with antibodies directed against α-synuclein aggregates, or ubiquitinated proteins. MSA currently comprises subtype MSA-C and MSA-P, based on a set of predominant symptoms including autonomic failure, and distinct sites in the brain with relative selective vulnerability. The MSA-C is considered when the chief symptoms reflect cerebellar dysfunction, with the cerebellum and brainstem bearing the brunt of the degenerative process, which used to be referred to as olivopontocerebellar atrophy. The subtype MSA-P is considered when parkinsonism prevails, with the brunt of the changes involving the striatum (caudate nucleus, putamen, and globus pallidus) and the substantia nigra (pars compacta and pars reticulate), which used to be referred to as striatonigral degeneration. However, the overlaps of the symptoms and the main sites or systems of degeneration could be prominent as MSA progresses, despite the predominant involvement of either the cerebellar or the striatonigral system.

All patients had signed an informed consent form approved by the Columbia University Medical Center Internal Review Board. We excluded 4 out of 14 PSP patients due to the coexisting basal ganglia ischemic stroke, Alzheimer’s disease and cerebral amyloid angiopathy, and insufficient documentation. We also excluded 2 out of 15 MSA patients with insufficient documentation.

### Definitions in symptoms and signs recorded

We only recorded the symptoms and signs documented in >50% of the charts unless specified otherwise. Definitions of the terminologies are as follows. AAO: age at the first reported symptom considered attributable to PSP or MSA. Latency: the period between the onset of the specific symptom and the motor symptom onset. Disease duration: time between the age of onset and death. Falls: any fall related to the disease. Cognitive decline: perceived by the patient, the relatives or the physician, including reports of intellectual or functional decline, or the decline in MMSE (mini-mental status examination, normal scores cutoff ⩾26/30) or mMMSE (modified MMSE, normal score cutoff ⩾51/57) score. The total mMMSE score used in minority of the patients here was proportionally converted to commonly used MMSE score for quantitative comparison. Speech disturbance: any alteration in speech quality compared with that before the disease onset. Swallowing difficulty: any reported difficulty, particularly coughing to liquid or choking to solid food, or a formal abnormal swallowing evaluation. Supranuclear gaze palsy: restricted eye movement, with preserved vestibule-ocular reflexes on exam. Down-gaze palsy: restricted downward eye movement, with preserved vestibule-ocular reflexes on exam. Square wave jerk: inappropriate saccades that take the eye off the target, followed by a nearly normal intersaccadic interval (~200 ms), and then a corrective saccade that brings the eye back to the target with same speed. Clinically our cases were judged by a quick side-to-side globe movement of same speed in each direction with visual fixation. Pyramidal involvement: both brisk reflexes and pathologically extensor plantar or Hoffmann response(s). Autonomic dysfunction: either abnormal autonomic function testing or documentation of orthostatic hypotension, urinary incontinence, or any two of the following groups: urinary urgency or frequency; impotence; sweating abnormalities; skin color change; or severe constipation in need of medication on a regular basis. Urogenital dysfunction: urinary incontinence, or at least two of the following symptoms of impotence, urinary urgency or urinary frequency. Ataxia: any two of the following symptoms: ataxic gait (unsteady staggering gait with irregular pace and rhythm and usually broad base as well), dysmetric heel-knee-shin test or finger-to-nose test, broad base, titubation, persistent nystagmus, scanning speech, or unable to tandem walk. Frontal lobe dysfunction: apathy, lack of motivation, perseveration, withdrawal, personality change, or as suggested by neuropsychological tests. Response to levodopa: improvement in parkinsonism from the notes or Unified Parkinson’s Disease Rating Scale of >30% coincident with the introduction of levodopa or other medications for parkinsonism.

### Statistical analysis

Non-paired Student’s *t*-test was used for demographics or symptoms comparison between PSP and MSA. A *P*-value of 0.05 was viewed as significant. A linear Pearson correlation was applied to study the relationship between a specific symptom and disease duration.

## Results

### Demographics, clinical symptoms and signs

As presented in the Table 1, 10 PSP and 13 MSA patients (7 MSA-P and 6 MSA-C) were eventually studied, incidentally well matched in AAO, gender, and disease duration between two diseases. The final antemortem clinical diagnosis was correct in 70% of the PSP patients, all with down-gaze palsy, with the remaining 30% of patients being misdiagnosed (due to the lack of down-gaze palsy) as either MSA due to the presence of dysautonomia, ataxia and pyramidal involvement, corticobasal degeneration due to the presence of apraxia, alien hand phenomena, dystonia and abnormal positron emission tomography imaging, or frontotemporal lobe dementia parkinsonism syndrome due to the presence of frontal lobe dysfunction. The final antemortem clinical diagnosis was correct in 77% of the MSA patients. The remaining 23% (3 patients, all MSA-P) of patients were misdiagnosed as atypical or vascular parkinsonism, all presenting with parkinsonism but without ataxia, with no dysautonomia in life in one of them, and delayed dysautonomia at year 3 after disease onset in another two.

The common presenting symptoms were falls (40%), parkinsonism (40%), and cognitive/behavioral change (30%) for PSP; and parkinsonism (46%, all MSA-P), dysautonomia (38%, 2 MSA-P and 3 MSA-C), and ataxia (31%, all MSA-C) for MSA. Falls within 1 year of the disease onset was similarly seen in 50% of PSP and 38% of MSA. The latency to the first fall was similar in PSP and MSA (1.7±1.8 and 2.7±2.3 years, respectively, *P*=0.278).

Down-gaze palsy and square wave jerks were present in most of the PSP patients (70 and 90%, respectively), but much less common in MSA (8 and 23%, respectively). Dysautonomia was present in at least 80% of the PSP patients during the disease course, but involved only urogenital dysfunction without orthostatic hypotension, and none was present before the onset of the motor symptoms. Dysautonomia was present at motor symptom onset in 38% of the MSA patients, and in 46, 62, and 77% of the patients 1, 3 and, 5 years, respectively, afterwards. Interestingly, 23% of the MSA patients even had dysautonomia as early as 3 years preceding the onset of the motor symptoms, and 15% of the MSA patients never had dysautonomia in life, despite being assessed in the last year of their life.

Dystonia occurred in at least 50% of the patients with PSP, and 46% of MSA, with anterocollis being seen only in MSA patients. Ataxia occurred in at least 50% of the patients with PSP, on average 4.6 years after the motor symptom onset, and in at least 61% (6 MSA-C, 2 MSA-P) of the patients with MSA, with ataxia as the presenting features in all MSA-C. Pyramidal involvement was seen in at least 30% of the patients with PSP and MSA (both MSA-C and MSA-P). Apraxia occurred in at least 50% of the patients with PSP, 20% with alien limb phenomena and 10% with cortical sensory loss. There was no difference in the latency of first fall (1.7±1.8 vs. 2.7±2.3 years, *P*=0.278), dysarthria (2.7±2 vs. 2.3±2.5 years, *P*=0.724), dysphagia (3.5±1.7 vs. 4.1±2.8 years, *P*=0.612), or the percentage of the patients with freezing of gait (50 vs. 46%, *P*=0.6217) between PSP and MSA.

Laryngeal stridor was present in at least 30% of the MSA patients (1 MSA-P and 3 MSA-C) on average 4.8 years after motor onset, and all died 2–5 years later, including one who had tracheostomy. Myoclonus was present in at least 38% patients of MSA (3 MSA-P and 2 MSA-C), even in the absence of dopaminergic medications in some patients.

The average MMSE score in PSP was mildly impaired at their first visit of 2.7 years on average after the disease onset. Consistently, frontal lobe dysfunction was documented in 70% of the PSP patients. Cognition was normal to mildly impaired in MSA, as assessed on average of 5 years after motor onset.

### Response of the parkinsonism to levodopa treatment

Response of the parkinsonism to levodopa treatment was seen in at least 30% of the PSP patients, all of whom had resting tremor, at 450–2000 mg daily in the first 4–7 years of disease; and in at least 54% of the patients with MSA (4 MSA-P, and 3 MSA-C), at 200–1300 mg daily in the first 2–10 years.

### Correlation between disease duration and important clinical features

**Figure 1:**
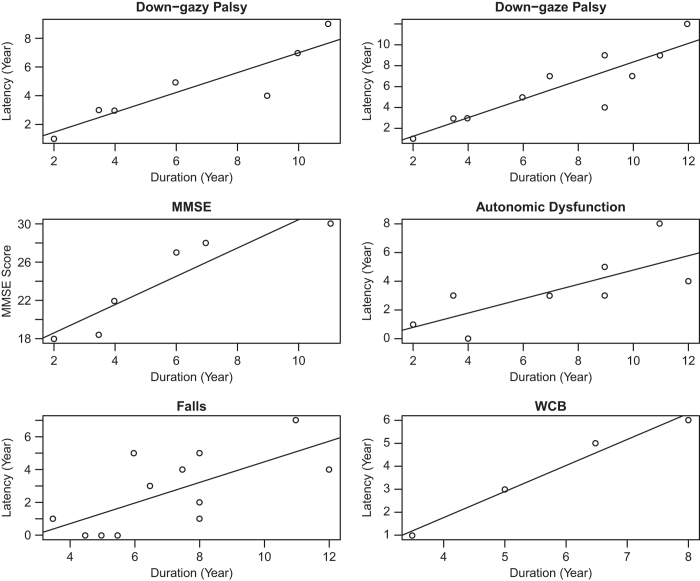
Correlation between disease duration and important clinical features. The correlation between disease duration and the latency of down-gaze palsy (top left panel, in 7 patients with down-gaze palsy before death, with Pearson’s correlation coefficient *r*=0.902, *P*=0.005; and top right panel, in 10 patients including the 3 patients without down-gaze palsy in life, taking the duration of the disease as the latency in these 3 patients, with *r*=0.8893, *P*=0.001), MMSE score at first visit (on average the first 2.7 years after disease onset, mid left panel, *r*=0.920, *P*=0.009) and the latency of dysautonomia (mid right panel, *r*=0.752, *P*=0.031) in PSP; and the latency of falls (bottom left panel, *r*=0.675, *P*=0.016) and WCB (wheel chair bound, bottom right panel, *r*=0.990, *P*=0.010) in MSA.

The early manifestation of down-gaze palsy (in seven patients with down-gaze palsy before death, Pearson correlation coefficient *r*=0.902, *P*=0.005), urogenital dysfunction (*r*=0.752, *P*=0.031), or poor MMSE score at their first visit (on average the first 2.7 years, *r*=0.920, *P*=0.009) in PSP, and the early presence of the first fall (*r*=0.675, *P*=0.016) or wheelchair confinement (*r*=0.990, *P*=0.01) in MSA were all associated with a short disease duration or rapid progression (Figure 1, all panels except the top right panel). The latency of the down-gaze palsy was still associated with disease duration in PSP even when we included all 10 patients (counting the disease duration as the latency for the three patients without down-gaze palsy before the death, *r*=0.8893, *P*=0.001) (Figure 1, top right panel). There was no correlation between the disease duration and AAO, and the latency of speech or swallowing dysfunction in PSP and MSA.

### Brain imaging

Brain magnetic resonance imaging (MRI) was all reported to be normal or to show mild diffuse atrophy in eight PSP patients, one with midbrain atrophy. There was no reported cerebellar atrophy in PSP with cerebellar ataxia patients based on radiologists’ report, though the brain MRI was obtained 2 years earlier (2.6±1.1) than the onset of the ataxia (4.6±1.5). Cerebellar atrophy was reported in 90% of the nine MSA patients, with two pontine atrophy (1 MSA-C and 1 MSA-P) and one hot cross bun sign.

## Discussion

Our study not only confirmed most of the previously reported clinical features of PSP and MSA, but also for the first time found that down-gaze palsy has a prognostic value in PSP progression (Pearson correlation coefficient *r*=0.902, *P*=0.005). Our novel finding is supported clinically by the fact that the PSP-RS with early down-gaze palsy (usually in 2 years after the disease onset) has a more rapid disease progression and shorter disease duration (on average 5.9 years) than PSP-P, which has late onset or even no down-gaze palsy with longer disease duration (on average 9.1 years).^8,10^ Indeed similarly, our three PSP patients who did not have down-gaze palsy (therefore were all misdiagnosed) had parkinsonism and longer disease duration (9.3±2.5 years) compared with the seven PSP patients with down-gaze palsy (6.5±3.5 years), though not statistically different due to the small sample size, with one having additional unilateral resting tremor and good response to levodopa treatment, one having essential tremor, and the other having corticobasal syndrome. Our novel finding could also be explained pathologically by the fact that most PSP patients started with globus pallidus interna, subthalamic nucleus, and substantia nigra par compacta and gradually extended rostrally to the frontal lobe, parietal lobe, and other regions, and caudally to the brainstem and cerebellar dentate nucleus.^9^ Brainstem is suggested to control supranuclear eye movements.^15^ Hence, the early occurrence of supranuclear palsy indicates a rapid progression of the disease and short survival duration. Similarly, we could also explain the association of the early occurrence of the cognitive dysfunction with short disease duration, consistent with a previous report,^10^ though MMSE may not be the best test to assess cognitive dysfunction in both disorders. Our finding could help predict the PSP progression, which is very important for both family planning and disease management.

Urogenital dysfunction is not uncommon in our PSP cases, as reported elsewhere.^6,16,17^ Interestingly, the latency of the urogenital dysfunction is associated with the disease duration in our patients. The urogenital dysfunction might well represent frontal lobe dysfunction in PSP, given the lack of orthostatic hypotension and the absence of urogenital dysfunction before the disease onset in our study and another report.^16^ Dysautonomia could occur as early as 3 years before the motor onset in our MSA patients. Interestingly, 15% of the MSA patients had no documented dysautonomia, even assessed in their last year of life, consistent with another report,^18^ which makes the diagnosis of MSA more challenging. Indeed, 8% of the patients missed the diagnosis partially due to the absence of dysautonomia in our study. Worth noting that one cannot absolutely claim that in some cases MSA patients did not have dysautonomia but rather that this was not clinically reported. It is conceivable that in some cases autonomic dysfunction might be mild and only detected instrumentally.

Ataxia used to be listed in the exclusion criteria of PSP.^2^ However, it is not surprising that ataxia was also found in at least 50% of the PSP patients, but at a later disease stage of 4.6 years on average after the disease onset, as the dentate nucleus in the cerebellum is involved later on as the disease advances caudally, different from that in MSA-C with ataxia as a presenting feature. There was no reported cerebellar atrophy in PSP patients with cerebellar ataxia based on radiologists’ report, which is different from that in MSA, as cerebellar atrophy in brain MRI is commonly seen in our MSA-C and MSA-P patients. The obtaining of brain MRI 2 years earlier than the onset of the ataxia in our patients might contribute to the lack of association between ataxia and imaging change. Ataxia has been reported in another study in PSP.^12^ It may be plausible to consider an additional type of PSP, PSP-C (cerebellar type), for those with a prominent feature of ataxia.

Apraxia, another sign that used to be in the exclusion criteria of PSP,^2^ was also found in at least 50% of our PSP cases, 20% even with alien hand phenomena, and 10% with cortical sensory loss, which most likely is PSP-corticobasal syndrome,^11^ with the morphology of PSP instead of corticobasal degeneration.^19^ There was no apraxia in MSA, as expected, which helps to differentiate it from PSP.

The presence of limb dystonia in PSP and anterocollis in MSA cases is also consistent with a previous report.^20^

The response of parkinsonism to levodopa was seen only in PSP with resting tremor, consistent with the notion of PSP-P, who has tremor and parkinsonism with good response to dopaminergic medication treatment.^8^ The percentage of the patients responsive to dopaminergic medication in PSP and MSA is similar to that reported before,^6,10^ indicating that dopaminergic medication is worth trying in these diseases, particularly in PSP with resting tremor. A poor prognosis was found in MSA cases who had early falls and wheelchair confinement, consistent with a previous report.^10^

Our study has its unique strength and novelty. First, all the patients had been followed clinically in a single tertiary movement disorder center for several years until autopsy, with good methodological and expertize uniformity. Second, the patients with other concomitant brain diseases were excluded to avoid confounding clinical features. Third, the AAO, gender, and disease duration were all incidentally well matched in PSP and MSA, which make a fair comparison between PSP and MSA possible. Fourth, we studied the diagnostic milestones of PSP (down-gaze palsy) and MSA (dysautonomia), and other important symptoms in temporal evolution for the diagnostic and prognostic value of the symptoms. Most importantly, we for the first time found the novel linear prognostic value of the down-gaze palsy latency in PSP progression, which would have significant clinical implications.

Our study also has its limitations. The sample size is small and information is missing for some patients. We might have a selection bias, as only the atypical cases were sent to our tertiary center, which is probably why our MSA cases had older than usual AAO. The retrospective reviewing of the charts could have less comprehensive information. The assessment of the ataxia in PSP might not be accurate given the presence of postural instability and the complexity of the ataxia in this retrospective study. The MRI results were taken from radiology reports without being read by us, which could have a false negative report. The dysautonomic symptoms could be confounded by the comorbidities of diabetes, enlarged prostate in men or stress incontinence in woman, which may not have been formally diagnosed or well documented. A further study with large size in a prospective cohort would help to corroborate our results in the future.

In conclusion, our pathologically verified PSP and MSA patients followed for years by movement disorder specialists in a tertiary center provide valuable clinical features for the diagnosis and prognosis of PSP and MSA. We not only verified many previously reported characterizations, but also for the first time found the prognostic value of the down-gaze palsy latency in PSP progression, which would have significant clinical implications.

## Figures and Tables

**Table 1:** Clinical features of pathologically confirmed PSP and MSA patients

*Disease*	*Cases*	*PSP (a total of 10 patients)*	*Cases*	*MSA (a total of 13 patients)*
Gender	10	6M/4F	13	8M/5F, 7P/6C
AAO	10	64.5±8.6 (46–75 years)	13	66.3±6.9 (56–80 years)
Duration	10	7.4±3.4 (2–12 years) (6.5±3.5 with down-gaze palsy, 9.3±2.5 w/o it)	13	7.5±2.8 (3.5–12 years)
Last diagnosis	10	7 (70%) PSP; missed 30% (as MSA, CBD, FTDP, no down-gaze palsy)	13	10 (77%) MSA; missed 23% (as atypical or vascular parkinsonism),
Onset symptom	10	4 (40%) parkinsonism, 4 (40%) fall, 3 (30%) cognitive/behavioral change	13	6 (46%) parkinsonism (all P), 38% (2P 3C) dysautonomia, 4 (31%) ataxia (all C)
Falls	10	4 (40%) onset, 5 (50%) in 1 year, 7 (70%) in 2 years, 9 (90%) in 3 years	13	3 (23%, 2C 1P) onset, 5 (38%) in 1 year, 11 (85%) in 5 years
Down-gaze palsy	10	7 (70%) in the course, 2 (20%) with additional horizontal gaze palsy	13	1 (8%) in the course (1C)
SWJ	10	9 (90%) in the course	13	3 (23%) in the course (1P 2C)
Dysautonomia	8	8 (at least 80%), only urogenital dysfunction, no OH, none before MO	13	5 (38%) onset; 46, 62, 77% in 1,3,5 years; 23% before motor; 15% (2P) w/o in life
Dysarthria	10	10 (100%) in the course	13	10 (100%) in the course
Dysphagia	4	4 (at least 40%) in the course	8	8 (at least 62%) in the course
FOG	5	5 (at least 50%) in the course	9	6 (at least 46%) in the course
Apraxia	5	5 (at least 50%) in the course	7	None
Tremor	7	7 (at least 70%) in the course (3 RT with 2 unilateral RT, 3 AT, 2 PT)	13	9 (70%) in the course (AT 54% all P, PT 23% all P, RT 15% with 1P 1C)
Dystonia	6	5 (at least 50%) in the course	7	6 (at least 46%; 3P 3C) in the course
Ataxia	5	5 in the course (at least 50%), on average 4.6 year after MO	12	8 in the course (at least 61%, 6C 2P)
Pyramidal sign	9	3 in the course (at least 30%)	11	4 in the course (at least 30%, 3P 1C)
MMSE	6	6 (at least 60%), 23.5/30 at first test average 2.7 years after MO	13	12 normal/mild deficits by history or MMSE/mMMSE average 5.0 years after MO
Stridor	0	Not mentioned	8	4 (at least 30%; 1P 3C), all died 2–5 years later
Myoclonus	0	Not mentioned	5	5 (at least 38%; 3P 2C); 2 of them not on medications (1C 1P)
MRI	8	8 (at least 80%), normal or mild diffuse atrophy, one midbrain atrophy	9	8 (at least 61%, 6C 2P) cerebellar atrophy, pons atrophy (1C 1P) cross bun (1C)
L-dopa	8	3 (at least 30%) responsive, all with RT, 450–2000 mg/day for 4–7 years	12	7 (at least 54%, 4P 3C) responsive, 200–1300 mg/day, for 2–10 years

Abbreviations: AAO, age-at-onset; AT, action tremor; C, MSA-C; FOG, freezing of gait; FTDP, frontal-temporal lobe dementia with parkinsonism; MMSE, mini-mental status examination; MO, motor onset; MRI, magnetic resonance imaging; MSA, multiple system atrophy; OH, orthostatic hypotension; P, MSA-P; PSP, progressive supranuclear palsy; PT, postural tremor; RT, resting tremor; SWJ, square wave jerk; W/O, without. Notes: The number under ‘Cases’ is the total number of charts with that symptom recorded. The number next to it is the actual number of charts with that symptom present. ‘At least’ is used if not all the charts mentioned that symptom, with 10 for PSP or 13 for MSA as denominator.
